# *Hymenolepis diminuta*-based helminth therapy in C3(1)-TAg mice does not alter breast tumor onset or progression

**DOI:** 10.1093/emph/eoab007

**Published:** 2021-02-12

**Authors:** Scott Sauer, Dylan Beinart, Sade M B Finn, Sereena L Kumar, Qing Cheng, Shelley E Hwang, William Parker, Gayathri R Devi

**Affiliations:** Department of Surgery, Duke University Medical Center, Durham, NC 27710, USA

**Keywords:** breast cancer, evolutionary mismatch, hygiene hypothesis, mouse model, helminth therapy

## Abstract

**Background and objectives:**

An individual’s risk of breast cancer is profoundly affected by evolutionary mismatch. Mismatches in Western society known to increase the risk of breast cancer include a sedentary lifestyle and reproductive factors. Biota alteration, characterized by a loss of biodiversity from the ecosystem of the human body as a result of Western society, is a mismatch known to increase the risk of a variety of inflammation-related diseases, including colitis-associated colon cancer. However, the effect of biota alteration on breast cancer has not been evaluated.

**Methodology:**

In this study, we utilized the C3(1)-TAg mouse model of breast cancer to evaluate the role of biota alteration in the development of breast cancer. This model has been used to recapitulate the role of exercise and pregnancy in reducing the risk of breast cancer. C3(1)-TAg mice were treated with *Hymenolepis diminuta*, a benign helminth that has been shown to reverse the effects of biota alteration in animal models.

**Results:**

No effect of the helminth *H. diminuta* was observed. Neither the latency nor tumor growth was affected by the therapy, and no significant effects on tumor transcriptome were observed based on RNAseq analysis.

**Conclusions and implications:**

These findings suggest that biota alteration, although known to affect a variety of Western-associated diseases, might not be a significant factor in the high rate of breast cancer observed in Western societies.

**Lay summary:**

An almost complete loss of intestinal worms in high-income countries has led to increases in allergic disorders, autoimmune conditions, and perhaps colon cancer. However, in this study, results using laboratory mice suggest that loss of intestinal worms might not be associated with breast cancer.

## BACKGROUND AND OBJECTIVES

Many chronic diseases are associated with contemporary Western lifestyle factors, which were present at much lower levels or even absent prior to the industrial revolution [1]. Allergic conditions, autoimmune disorders and cardiovascular diseases fall into this category, with underlying causes, termed ‘evolutionary mismatches’, that are abundant in developed countries but are not prevalent or do not exist in hunter-gatherer societies [2]. These mismatches include inflammatory diets characterized by high fat and processed carbohydrates, sedentary lifestyles due to use of labor-saving technology [3] and widespread vitamin D deficiency due to indoor work environments. Evolutionary mismatches typically interact with individual genetics, creating a matrix of disease risk that is highly specific for each individual, yet associated with societal factors. Although chronic inflammation-associated conditions such as cardiovascular disease and allergies are most commonly considered to be evolutionary mismatches, some cancers are also known to be a result of evolutionary mismatch [4].

Breast cancer is the most frequent cancer among women, resulting in the death of >600 000 women worldwide in 2018 [5]. Substantial evidence indicates that the high rate of breast cancer in industrialized countries is due, at least in part, to evolutionary mismatch. Obesity, for example, is strongly associated with breast cancer [6–8], being linked with a 20–40% increase in the risk for receptor-positive postmenopausal breast cancer [8]. Obesity results from at least two evolutionary mismatches, sedentary lifestyles and Western diets, and affects >40% of US adults [9], but <5% of people in hunter-gatherer cultures [3]. In this context, we consider the hunter-gatherer state to be the environment of evolutionary adaptation [10], and thus, by definition, devoid of issues associated with evolutionary mismatches between our genetics and our current culture. That being said, it is acknowledged that many if not most current evolutionary mismatches affecting humans in high-income countries were introduced during the second industrial revolution, circa 1870-1914.

One such evolutionary mismatch that gained prominence following the second industrial revolution, the loss of complex eukaryotic symbionts from the ecosystem of the human body, is associated with a wide range of inflammatory diseases, including a variety of allergic disorders and autoimmune conditions [11–13]. Helminths and intestine-dwelling protists in particular have been all but eradicated by modern sanitation and food storage practices in high-income countries. The presence of these organisms is known to have a profound impact on immune function, decreasing pathologic, non-adaptive inflammation in humans and in a variety of animal models [14, 15]. Mechanisms by which helminths regulate immune function include generation of regulatory networks and production of anti-inflammatory molecules [16–21]. It might be hypothesized that reduction of inflammation could reduce the induction and progression of breast cancer, given that breast cancer is associated with inflammation [22] and that various inflammatory factors (e.g. obesity, alcohol consumption and sedentary lifestyle) are associated with breast cancer [22]. However, the role of inflammation in breast cancer is complex [22] and may depend strongly on the type of breast cancer [23]. Further, while the re-introduction of helminths has been shown to ameliorate colon cancer in an animal model of the disease [24], the effects of reintroducing helminths on breast cancer have not been evaluated.

In this study, we used the well-established C3(1)-TAg mouse model of breast cancer to probe the role of helminths in the development of breast cancer. Specifically, we tested the hypothesis that the presence of the rat tapeworm, *Hymenolepis diminuta*, might alter the onset and progression of breast cancer in the mice. *Hymenolepis diminuta* was selected because (a) it is currently being used for helminth therapy in humans, with apparently promising results [25–27], and (b) it has been shown to eliminate pathologic inflammation in laboratory mice [28]. The organism is benign and beneficial [29] in its native host, *Rattus norvegicus*, and fails to develop to maturity in both mice and humans. Thus, in both mice and in humans, exposure to the organisms must be repeated on a regular basis to maintain its therapeutic function.

Although several helminths are currently being used for therapeutic purposes in humans, no systematic search of an ideal candidate helminth has been conducted [30]. Nevertheless, work in the field suggests that a wide range of helminths, either cestodes or nematodes, work in a similar fashion to ameliorate chronic, pathologic inflammation [26, 31]. However, at the present time, *H. diminuta* is the only helminth that has been studied as a potential therapeutic agent in both humans [25–27] and in laboratory animals [28, 29, 32]. Further, given that not all helminths are beneficial [33, 34], it was important to select a helminth such as *H. diminuta* that has an established therapeutic benefit. Importantly, based on studies in animals [28, 29, 32] and in humans [25–27, 35], it is apparent that exposure to helminths that do not survive to sexual maturity in the GI tract are adequate for anti-inflammatory therapy, apparently reversing the results of loss of exposure to helminths. Given that these ‘non-colonizing’ helminths have several advantages when considering clinical use, including the ease of control of the organisms, *H. diminuta* is an excellent model for eventual clinical translation.

## METHODOLOGY

### Mice

All procedures utilizing laboratory animals were approved by the Duke University Institutional Animal Care and Use Committee. Four-week-old WT (FVB/NJ) female and FVB-Tg(C3-1-TAg)cJeg/JegJ transgenic male mice were obtained from Jackson Laboratories.

### Helminths

*Hymenolepis diminuta* cysticercoids (HDCs) were produced as previously described [25] using laboratory rats as primary hosts and grain beetles (*Tenebrio molitor*) as intermediate hosts. The HDC rather than eggs or the adult life stage are used for therapy in mammals since it is this stage that, when ingested, excysts and provides therapeutic effect [25]. After extraction from their insect hosts, four intact HDCs in 100 μl of saline were fed to mice via oral gavage once per week in the treatment arm. As a control, 100 μl of saline without HDCs were fed to mice via oral gavage once per week. The dosage of HDCs used was based on previous studies by Derek McKay’s lab in which 1–5 HDCs per mouse were used in a single dose with good therapeutic effect [28, 36]. In addition, it is known that mice eliminate HDCs within 7–10 days in a mast cell-dependent manner [37]. Given these parameters and given our observations that occasionally one or (rarely) two HDCs may be lost during transfer, apparently as a result of adhering to the pipette, four HDCs/mouse/week were selected for the treatment regimen.

### Study design

Female FVB/N F_0_ mice (*n* = 10) and male C3(1)-TAg F_0_ mice (*n* = 10) received weekly treatment with four HDCs/mouse. In addition, female FVB/N F_0_ mice (*n* = 10) and male C3(1)-TAg F_0_ mice (*n* = 10) received saline controls as listed in Table 1. Feeding continued until a humane endpoint was reached or until completion of weaning of their offspring. On Day 25, after the third exposure to HDCs, the 20F_0_ transgenic males were bred with the 20 FVB/N F_0_ female mice such that HDC-treated males were bred with HDC-treated females, and sham-treated males were bred with sham-treated females. The desired heterozygous genotype of F_1_ offspring [both male and female offspring possessing only one copy of the C3(1)-TAg transgene on the FVB/N background] were confirmed by the presence of SV40 transgene in DNA obtained from tail clippings as previously described [38]. At the time of weaning, 68F_1_ mice, 34 males and 34 females, possessing the C3(1)-TAg genotype were selected for study such that half of the male and female F_1_ mice where derived from HDC-treated parents, and the other half from sham-treated mice. Also at the time of weaning, F_1_ mice began receiving weekly treatments of 4 HDCs/mouse/week or saline until a humane endpoint was reached. Female animals were monitored twice per week and male mice were monitored once per week for palpable tumors. Of the 68 mice selected for study, 9 did not complete the study and were sacrificed following health-related humane endpoints relating to complications from oral gavage. Tumor volume (*L* × *W*^2^) was assessed with caliper measurements for length (*L*) and width (*W*) based on the orientation of the mouse (i.e. measurements made in the vertical plane will be classified as *L* and in the lateral plane as *W*) as described previously [39–42]. Mice were sacrificed by CO_2_ asphyxiation when they showed signs of morbidity or when tumors reach a volume of 2000 mm^3^.

**Table 1.** Experimental groups

Group	Weekly treatment	Number of mice
F_0_ sham exposure	100 μl saline	10 male and 10 female
F_0_ HDC exposure	4HDCs in 100 μl saline	10 male and 10 female
F_1_ sham exposure	100 μl saline	14 male and 15 female
F_1_ HDC exposure	4HDCs in 100 μl saline	17 male and 13 female

All groups, both male and female, in the F_1_ generation initially contained 17 mice. The number shown is the number that completed the study.

The design of this study included treatment of both F_0_ mice and F_1_ mice with helminths for two primary reasons. First, in female C3(1)-TAg mice, the development of breast tumors is rapid, with an average time to onset of <150 days. Thus, it was expected that early initiation of therapy would provide the greatest chance of success. Given that very young mice cannot tolerate oral gavage due to fragility, treatment of the parental generation was used as a method to potentially expose the animals to any putative beneficial effects of the therapy. It was anticipated that potential benefits of helminth therapy, if present, may be passed to pups from their parents either through epigenetic mechanisms (from either the sire or the dam), through the placenta during development *in utero*, or through ingestion of immune components in the dam’s milk. Evidence for therapeutic benefits to young pups from parental exposure has been observed in a rat model [29], but has not been evaluated in a mouse model. Nevertheless, it was thought that the best likelihood of finding a beneficial effect of the therapy would involve treatment of both the F_0_ mice and F_1_ mice.

A second reason for treatment of both F_0_ mice and F_1_ mice was that initial treatment of humans with HDCs can induce an initial inflammatory response that subsides over time [27]. Further, in mice, initial treatment with HDCs near the time of exposure to disease causing stimulus can be detrimental rather than beneficial, at least in a model of colitis associated cancer [43]. With this in mind, it was thought that the best likelihood of finding a beneficial effect would involve long-term treatment of animals, without the initial exposure to helminths at a time when disease progression might already be underway.

### Euthanasia and post-mortem assessment

At the time of sacrifice, all gross tumors were individually counted, measured as previously described to determine tumor volume and removed from the mouse. Total tumor weight was recorded. Tumor samples from female mice were submitted to Qiagen (Hilden, Germany) for RNA isolation and evaluation using RNA-Seq technology. The Qiagen Mouse Inflammation and Immunity Transcriptome panel (RMM-005Z, containing 489 genes) was utilized. Data were analyzed using the QIAGEN CLC Genomics Workbench with the ready-to use workflow for panel RMM-005Z.

### Statistical analyses

For statistical analysis of survival data, tumor latency and tumor growth, GraphPad Prism, Version 5.0 (GraphPad Software; San Diego, CA, USA) was utilized as described previously [44]. An alpha of 0.05 was taken to be significant, and the means ± standard errors are reported. A log-rank (Mantel-Cox) test was used to compare survival curves, and an unpaired Student’s *t*-test was used to compare latency and growth data. The *P* values for the RNA-Seq data were calculated by Qiagen based on a Student’s *t*-test of the replicate normalized gene expression values for each gene in the saline-treated and helminth-treated groups.

## RESULTS

### Effect of HCD-based helminth therapy on tumor-free graft survival

**Figure 1.**
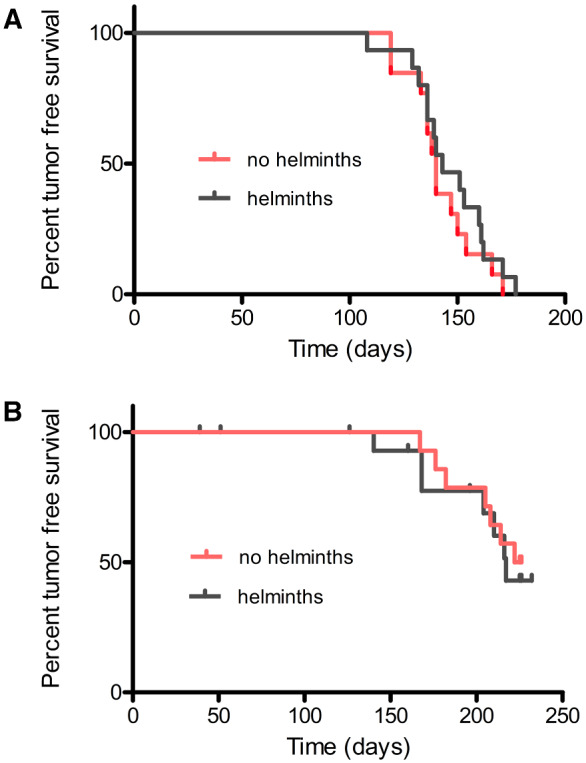
The effect of HDC-based helminth therapy on tumor free survival curves in female (**A**) and male (**B**) C3(1)-TAg mice No statistically significant difference between the treatment group and the controls was observed: *P*-values were 0.45 and 0.71 for females and males, respectively [log-rank (Mantel-Cox) test]

**Figure 2.**
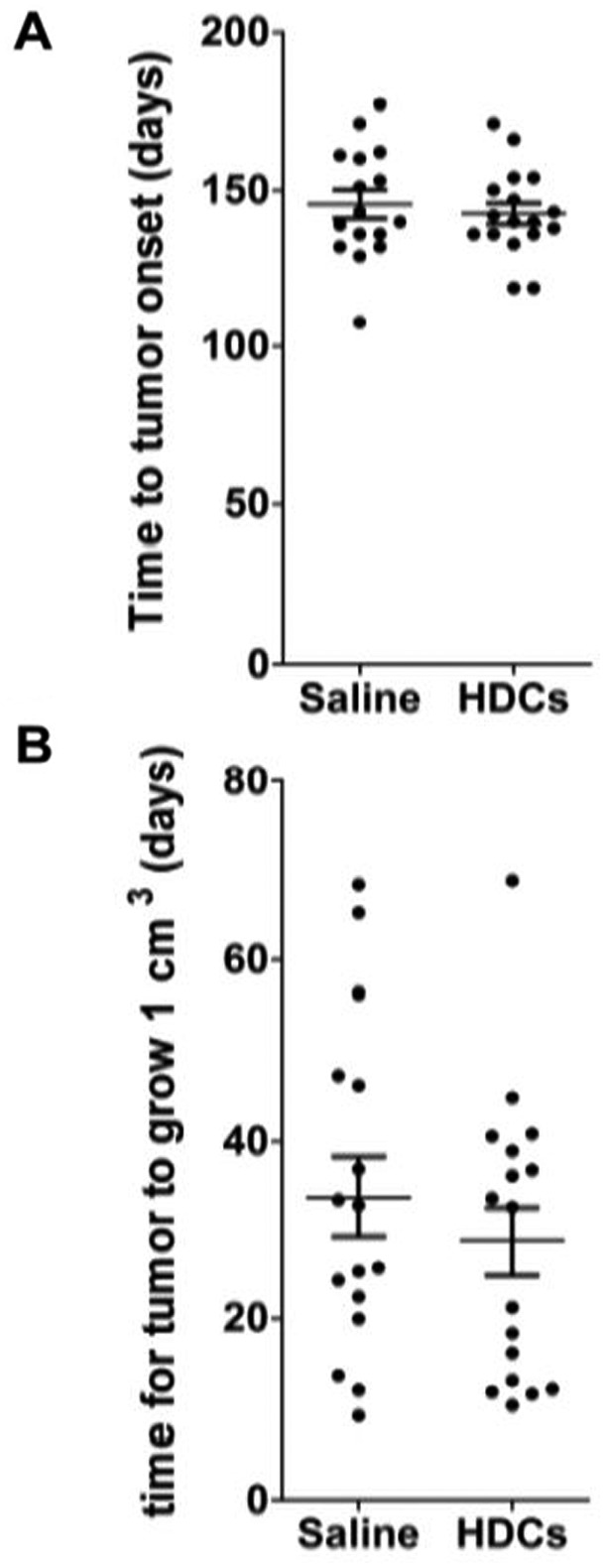
The effect of HDC-based helminth therapy on (**A**) time to onset of tumor and (**B**) growth rate of tumors in female C3(1)-TAg mice The time for the tumor to grow in size from the point of detection to 1.00 cm^3^ was used as a measure of growth rate. No statistically significant difference between the treatment group and the controls was observed: *P*-values were 0.59 and 0.47 for the time to onset and the growth rate, respectively (*t*-test)

Consistent with previous studies using C3(1)-TAg F_1_ mice [38], all 30 female C3(1)-TAg F_1_ mice evaluated in this study developed tumors within 180 days of age (Fig. 1A). Tumor onset was not significantly delayed or accelerated by the HDC-based treatment employed in this study (Figs. 1A and 2A). Further, following onset, tumor growth in female C3(1)-TAg mice was not measurably affected by HDC-based helminth therapy (Fig. 2B). By the age of 240 days, >50% of the 30 male C3(1)-TAg F_1_ mice had developed tumors, and the onset of tumors in these animals was also not affected by HDC-based helminth therapy (Fig. 1B).

### Effect of HCD-based helminth therapy on gene transcription in tumors

**Figure 3.**
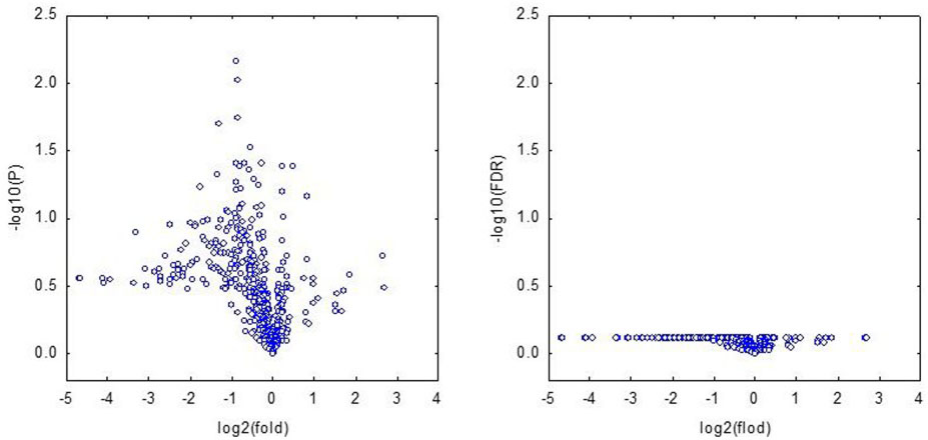
Volcano plots comparing gene expression between tumors from female C3(1)-TAg mice treated with and without HDC-based helminth therapy The pairwise comparison was performed using a Student’s *t*-test. None of the genes showed a significant difference in false discovery rate (FDR) between the two groups.

RNA-Seq analysis did not reveal any statistically significant differences between the transcriptome of tumors from HDC-treated female animals and the tumors of control female animals (Fig. 3). Tumors from male animals were not analyzed.

### Conclusions and implications

Despite the fact that helminth therapy can affect the onset and progression of colon cancer [24] and other inflammation-related diseases, including allergic [45] and autoimmune disorders [15, 18, 46], the results of this study do not provide any evidence suggesting that the presence or absence of helminths affects the induction or progression of breast cancers in humans. This finding may not be surprising, as evolutionary mismatches often affect one type of disease more so than another. For example, the role of vitamin D deficiency in neuropsychiatric disorders and autoimmune disease is well established [47], but apparently plays little role in allergic disease, at least in adults [48]. Further, breast cancer may be less driven by inflammation, per se, compared with other diseases that are affected by the presence of helminths, including colon cancer, allergy and autoimmune disease. Indeed, the current laboratory model for colon cancer involves exposure to inflammatory stimuli which create colitis. Similarly, allergy and autoimmunity are driven by aberrant immune responses. Breast cancer, on the other hand, is known to be driven by non-immune factors such as lifetime exposure to estrogen [49, 50].

Regardless of the results observed herein, studies in mice cannot be confidently extrapolated to humans. However, the C3(1)-TAg mouse model of breast cancer recapitulates the human condition well in studies addressing the role of evolutionary mismatch in breast cancer. For example, in C3(1)-TAg mice, exercise reduces the number of palpable tumors by 70% [51], recapitulating one role of evolutionary mismatch in human breast cancer. Although obesity, a risk factor for breast cancer, is induced by evolutionary mismatches of both diet and physical activity, it is the lack of physical activity that is most clearly connected with breast cancer in humans [52]. For example, in a 2000–2003 study of breast cancer cases in Poland, a 20% lower rate of breast cancer was observed in the quartile of subjects with the greatest reported lifetime physical activity compared with the quartile with the lowest level of activity [53]. The same study also found a 40% reduction in breast cancer rates in individuals reporting heavy physical work.

The role of Western diets, the other major evolutionary mismatch contributing to obesity in developed countries, has also been associated with breast cancer in some studies. However, the consumption of red meat is the only particular dietary factor that has been implicated in breast cancer [54, 55]. Although the consumption of red meat was probably not rare in hunter-gatherer societies, the difference between domestic livestock and wild game may be a factor, although this idea remains unexplored. In addition, it is thought that cancer cells, by their nature, may be unable to adapt to the fasting state [56]. As such, the lack of fasting in Western culture compared with hunter-gatherer societies [57, 58] may constitute an evolutionary mismatch, leading to an increase in a variety of cancers, including breast cancer [56]. With this in mind, studies may be warranted addressing the role of diet in the development of breast cancer in the C3(1)-TAg mouse model.

As another example of how the C3(1)-TAg mouse model of breast cancer recapitulates the role of evolutionary mismatch in human breast cancer, pregnancy causes reversal of preneoplastic mammary lesions in the mice [59]. Not generally considered an evolutionary mismatch, decreased rates of childbearing and breastfeeding in developed countries qualify as an evolutionary mismatch when considering human breast cancer [60]. Decreased parity, characteristic of Western cultures, has long been associated with breast cancer. In 1713, Ramazzini [61], considered to be the father of occupational health, noticed an association between breast cancer and religious orders requiring celibacy. More recently, a study comparing nuns with women not bound to celibacy found the probability of death from breast cancer for nuns between 70 and 79 years old to be almost double that of an age-matched control group [62]. Further, several studies have shown that women who have fewer uninterrupted menstrual cycles, more children and breastfeed more are less likely to get certain common subtypes of breast cancer. For example, an international group analyzing 47 independent studies from 30 countries with a total of >150 000 women found that for every 12 months a woman breastfeeds, the risk of breast cancer was reduced by 4.3%. For every birth, the risk decreased by 7% [63]. The authors conclude that breast cancer in high-income countries would be reduced by ∼40% if women had the average number of births and duration of breastfeeding that was prevalent in developing countries until recently.

The fact that the C3(1)-TAg mouse model of breast cancer recapitulates the role of the two major evolutionary mismatches known to be important in human breast cancer lends credence to this study. Further, the study was sufficiently powered, with 13–17 animals per group, to detect biologically significant effects and used a sufficient number and type of helminth to effectively modulate inflammation in mice [28]. In addition, the duration of treatment covered the entire lifespan of the animals since immune components from treated parents would have affected growth from conception to weaning. With these factors in mind, we believe that the negative results obtained in this study have merit. However, breast cancer is a heterogenous disease, and ‘biota alteration’, characterized by loss of helminths in modern, high-income countries, may affect one type of breast cancer, but not another. In addition, this study does not address the impact of other biota-associated factors in Western society that might affect the incidence and progression of breast cancer, such as the increased prevalence of contagious disease due to international travel and urban dwelling, changes in the gut microbiota as a consequence of changing diet or changes to the skin microbiota as a consequence of frequent bathing and loss of contact with the soil. Such studies may be worthwhile, with breast cancer being associated with particular viral infections [64], alterations in the microbiota [65] and even chronic inflammatory conditions of the upper respiratory tract [66].
